# Nanolithographic Fabrication Technologies for Network-Based Biocomputation Devices

**DOI:** 10.3390/ma16031046

**Published:** 2023-01-24

**Authors:** Christoph R. Meinecke, Georg Heldt, Thomas Blaudeck, Frida W. Lindberg, Falco C. M. J. M. van Delft, Mohammad Ashikur Rahman, Aseem Salhotra, Alf Månsson, Heiner Linke, Till Korten, Stefan Diez, Danny Reuter, Stefan E. Schulz

**Affiliations:** 1 Center for Microtechnologies, Chemnitz University of Technology, 09107 Chemnitz, Germany; 2 Department Nano Device Technologies, Fraunhofer Institute for Electronic Nano Systems (ENAS), 09126 Chemnitz, Germany; 3 Research Center for Materials, Architectures and Integration of Nanomembranes (MAIN), Chemnitz University of Technology, 09126 Chemnitz, Germany; 4 NanoLund and Solid State Physics, Lund University, 22100 Lund, Sweden; 5 Molecular Sense Ltd., Liverpool L36 8HT, UK; 6 Department of Chemistry and Biomedical Sciences, Linnaeus University, 39182 Kalmar, Sweden; 7 B CUBE—Center for Molecular Bioengineering and Cluster of Excellence Physics of Life, Technische Universität Dresden, 01307 Dresden, Germany; 8 Max Planck Institute of Molecular Cell Biology and Genetics, 01307 Dresden, Germany

**Keywords:** nanotechnology, electron-beam lithography, network-based biocomputation, microfluidics, molecular motors

## Abstract

Network-based biocomputation (NBC) relies on accurate guiding of biological agents through nanofabricated channels produced by lithographic patterning techniques. Here, we report on the large-scale, wafer-level fabrication of optimized microfluidic channel networks (NBC networks) using electron-beam lithography as the central method. To confirm the functionality of these NBC networks, we solve an instance of a classical non-deterministic-polynomial-time complete (“NP-complete”) problem, the subset-sum problem. The propagation of cytoskeletal filaments, e.g., molecular motor-propelled microtubules or actin filaments, relies on a combination of physical and chemical guiding along the channels of an NBC network. Therefore, the nanofabricated channels have to fulfill specific requirements with respect to the biochemical treatment as well as the geometrical confienement, with walls surrounding the floors where functional molecular motors attach. We show how the material stack used for the NBC network can be optimized so that the motor-proteins attach themselves in functional form only to the floor of the channels. Further optimizations in the nanolithographic fabrication processes greatly improve the smoothness of the channel walls and floors, while optimizations in motor-protein expression and purification improve the activity of the motor proteins, and therefore, the motility of the filaments. Together, these optimizations provide us with the opportunity to increase the reliability of our NBC devices. In the future, we expect that these nanolithographic fabrication technologies will enable production of large-scale NBC networks intended to solve substantially larger combinatorial problems that are currently outside the capabilities of conventional software-based solvers.

## 1. Introduction

Conventional computers solve problems sequentially, which limits their ability to compute combinatorial problems such as network routing, design of error-free computer chips and software, calculation of efficient use of resources in industrial processes, or prediction of protein folding. The challenge is the exponential increase in the necessary number of calculations in relation to the size of the system. Even relatively small combinatorial problems can overwhelm a conventional, sequentially operating computer with the number of arithmetical operations required to solve the problem. Alternative parallel computing approaches, such as DNA-computers [1] (DNA: desoxyribonucleic acid) or quantum computers [2], could in principle solve such problems much more efficiently. However, these methods have so far not been scalable in practice.

Another parallel computing approach is networked-based computing with biological agents [3], also known as network-based biocomputation (NBC). An NBC system is a physical, graphical representation of an instance of a mathematical problem that can be explored by a large number of autonomous agents, i.e., motile physical objects, e.g., cytoskeletal filaments, searching independently for a solution. In the case of NBC, motile physical objects can be represented by self-propelled microorganisms [3] or motile (yet passively guided) cytoskeletal filaments driven by localized molecular motors [4]. For different types of agents and molecular motors [3,4,5,6,7,8], there are various run modes and scaling parameters [9].

Hardware fabrication is related to scale-bridging manufacturing schemes that are adapted from the established manufacturing methodology in microelectronic device fabrication, including aspects of nanotechnology and schemes for heterogeneous systems integration [10].

To realize appropriate NBC networks with channel diameters in the sub-micrometer regime, various nanolithographic fabrication methods are possible: nanoimprint lithography, interference lithography, or projection lithography. However, electron-beam lithography (EBL) stands out as it combines both performant patterning capabilities on large areas (e.g., 200 mm wafers) and feature sizes down to sub-micrometers. 

In this paper, we present an optimized and scalable fabrication technology for NBC networks consisting of channels (down to 300 nm in width) that provide a combination of physical and chemical guiding of the cytoskeletal filaments, propelled by biomolecular motors localized in the channels. The basis for the work is the NBC nanofabrication workflow put forward in Ref. [4]. However, that workflow had certain shortcomings with respect to scalability. It is here improved toward a reliable nanolithographic wafer-level technology that also enables the fabrication of a large number of NBC devices and an increase in the NBC network sizes to up to 200 mm by optimizing the employed materials and the process flows. As a first technological challenge, the overall nanofabrication technology is revised to enable the processing of thin gold films, providing very smooth gold surfaces down to 20 nm surface roughness on the floors of the nanofluidic channels. Additional engineering challenges that are addressed comprise the optimization of individual etching processes aiming to minimize the surface roughness on nanochannel sidewalls and to further adapt the samples for a subsequent selective bio-functionalization of parts of the channels and junctions. 

Overall, we describe an optimized fabrication concept for network-based biocomputing devices, which inherently enables their upscaling in size and pattern-to-pattern individualization to solve various instances of combinatorial problems. We thus demonstrate that nanolithography is a key technology for NBC as it will lead to high flexibility and fast turnarounds for the implementation of different network layouts, extending its capabilities to larger mathematical problems. 

## 2. Fundamentals of Network-Based Biocomputation (NBC): A Brief Description

An NBC device in general comprises a physical, functionally designed computing network with a pool of possible solutions that require a sufficient supply of biological agents to explore all possible outcomes [9]. It relies on the accurate guiding of biological agents ensured by a combination of physical and chemical guiding of the molecular-motor-propelled agents along nanofabricated channels.

**Figure 1:**
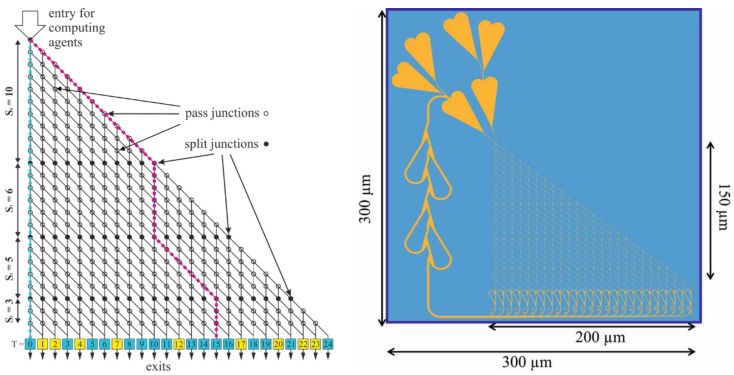
Schematic graphical representation of an NBC network (**left**) and the layout of a “real fluidic” NBC nanochannel network encoding the SSP instance {10, 6, 5, 3} (**right**). Left: Exits labeled in yellow and blue represent incorrect and correct solutions, respectively. Right: Layout encoding the SSP instance {10, 6, 5, 3} for both the microtubule-kinesin and the actin-myosin NBC systems, respectively. Areas (channels, loading zones, and feedback loops) accessible to the motile cytoskeletal filaments (microtubules, actin) are indicated in yellow; walls and inaccessible areas are indicated in blue.

NBC uses biomolecular agents, such as cytoskeletal filaments driven by biomolecular motors inside networks of micro- and nanofluidic channels, acting as programmable modules for mathematical problem-solving [5,11]. Biochemical processes in the human body inspire the storage and manipulation of data. The instance of the mathematical problem is coded into a channel network that can be fabricated by conventional semiconductor nanotechnology. It comprises (cf. Figure 1 right) initial loading zones for the filaments and confined channels, junctions (split junctions, pass junctions), detectors, and further architectural elements for computational processing [4]. The biological agents act as motile agents which are labeled or tagged with molecular flags, e.g., from DNA, nanoparticles, quantum dots, fluorescent molecules, and other building blocks [12].

### 2.1. Working Principle of NBC

In the field of network-based biocomputation, it has been proven that wafer-level NBC devices can successfully encode classical non-deterministic polynomial-time complete (“NP-complete”) problems, such as the exact-cover (ExCov) problem [13] and the satisfiability problem for three literals (3-SAT) [6], with details of the fabrication workflow touched on only briefly. In this paper, we report on a convergent optimized fabrication technology for NBC devices on the wafer level, illustrating the operation of a biocomputation network that encodes the subset-sum problem (SSP). The SSP is a well-known NP-complete problem in computer science, especially in cryptography and complexity theory [14]. It asks whether a target sum *T* can be reached by summing up any combination of integers from a set *S*.

In order to solve mathematical problems by biocomputation approaches, a problem is usually encoded into a graphical modular network with nanoscale channels. The network is embedded into a nanofabricated planar device (see Figure 1), which can be explored by biomolecular motor-powered filaments (or other types of agents). This graphical network consists of split junctions, which are responsible for distributing the cytoskeletal filaments evenly to all possible paths through the network, and pass junctions responsible for encoding the values of the numbers in the SSP [4]. 

### 2.2. NBC Network Layout

Figure 1 (left) shows a graphical representation of the computation network for the SSP instance {10, 6, 5, 3}. The agents enter the network from the top-left corner. Filled circles represent split junctions where it is equally probable that agents continue straight ahead or turn. Empty circles represent pass junctions where agents can only continue straight ahead. Moving diagonally down at a split junction corresponds to adding that integer (i.e., numbers 10 plus 5 for the purple example path in the network in Figure 1 (left). The actual value of the integer, potentially added at a split junction, is determined by the number of rows of pass junctions up to the next split junction or exit. The exit numbers correspond to the target sums *T* (potential solutions) represented by each exit; correct results for this particular set {10, 6, 5, 3} are labeled in blue, and incorrect results (where no agents should arrive) are labeled in yellow.

The layout for a computational network encoding the specific SSP instance {10, 6, 5, 3} is given in Figure 1 (right). The design of network-based biocomputation circuits for the computational solution of instances of mathematical problems, such as the exact-cover problem, requires a translation of the problem into a geometric grid that is manufacturable as a pattern (network) of microfluidic channels by contemporary high-throughput nanofabrication techniques [15,16]. The channels need to allow agents, such as cytoskeletal filaments, in large quantities to travel along defined trajectories and, thus, decode the instance of the mathematical problem repetitively.

## 3. Nanofabrication Technologies for NBC

Reliable guiding of the cytoskeletal filaments (the biocomputing agents) through the network is based on two essential physical requirements (cf. Ref. [17]): (I) Chemical guiding where only the channel floors have functional motor proteins. The walls do not have any active motors, and thus, the filaments are only able to attach to the channel floors. (II) Physical guiding where the channel topography provides physical confinement. This means that the filaments cannot escape the barrier of the walls and are instead forced to follow the channels. The combination of chemical and physical guiding results in very reliable guiding of the filaments in the desired direction [11,18].

Two different types of motor proteins and their associated filaments were investigated during the experiments:

1. The microtubule-kinesin system, using the biomolecular motor protein kinesin-1, which is robust in artificial environments and propels cytoskeletal microtubules as motile agents (MT) (cf. Refs. [19,20,21]).
2. The actin-myosin system, using the motor protein myosin-II, which is a fast motor that is readily available from skeletal muscle to propel cytoskeletal actin filaments as the motile agents (cf. Refs. [22,23]).

The fabrication of network devices was done using technology workflows based on the established manufacturing methods of microsystems, also covering nanotechnological aspects [17,24]. This results in a network of nanofabricated guiding channels, including split and pass junctions. The biological functionalization and necessary surface treatments needed for motility experiments were performed for two motor-protein/agent systems: the microtubule-kinesin [20] and the actin-myosin systems [23].

### 3.1. Microtubule-Kinesin System 

The biocomputational network used for the microtubule-kinesin system consisted of a network of SiO_2_ channels, with a Au floor, forming a system of hierarchical structures of pass/split junctions. The SiO_2_ was silanized with 2-[Meth-oxy(polyethyleneoxy)propyl]trimethoxysilane (PEG), such that the motor proteins (kinesin-1) could only adsorb on the Au floor and not on the surrounding SiO_2_ walls.

**Figure 2:**
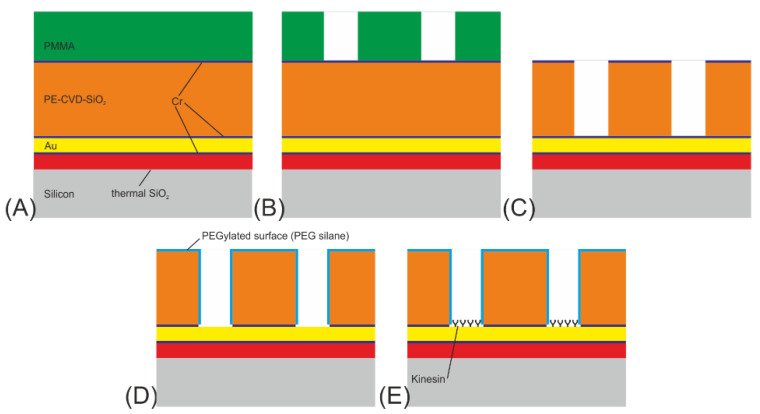
Process flow for NBC nanochannel networks for the microtubule-kinesin system: (**A**) growth of 100 nm thermal SiO_2_, sputter deposition of 10 nm Cr, 100 nm Au, 10 nm Cr, followed by PE-CVD of 500 nm SiO_2_ and sputter deposition of 10 nm Cr hard mask and finally spin coating of 400 nm PMMA. (**B**) E-beam-lithography for patterning the resist. (**C**) Reactive ion etching of Cr and SiO_2_. (**D**) Plasma etching of Cr for removing it from the top surface and the bottom of the channel followed by PEGylation of silicon oxide. (**E**) Functionalization of the gold at the channel floors by kinesin-1 motor proteins.

The network structures were fabricated on a standard 150 mm, single-side polished silicon wafer (625 µm thick) in five steps (see Figure 2):

1. The initial layer on the Si-substrate was a 100 nm thick SiO_2_ diffusion barrier layer made by dry thermal oxidation, carried out under an oxygen atmosphere with 3% HCl. An in situ sputter deposition followed, in which a 10 nm Cr adhesion layer, a 100 nm Au layer, and finally a 10 nm Cr layer were deposited onto the SiO_2_ diffusion barrier layer. In addition, a 10 nm thick Ti layer was investigated as an adhesion layer instead of the Cr layer. Next, a 500 nm SiO_2_ layer was deposited using plasma-enhanced chemical vapor deposition (PE-CVD) at 300 °C. Finally, a 10 nm Cr layer acting as a hard mask for the SiO_2_ patterning was sputter-deposited onto the surface (see Figure 2A).
2. The wafer was subsequently spin-coated with PMMA (ALLRESIST AR-P 679.04), a positive-tone electron-beam resist, to a resist thickness of 400 nm and pre-baked at 180 °C for 5 min (see Figure 2a). For the exposure, a 50 kV e-beam lithography system (Vistec SB254) was used at a dose of 650 µC/cm^2^. The PMMA was developed for 60 s at room temperature in a solution of one part methyl isobutyl ketone (MIBK) and three parts of isopropanol (IPA), rinsed with IPA, and flushed in a conductance-controlled bath of deionized (DI) water. In the final step, all remaining surface humidity was removed in a commercial dryer. This left the wafer with a resist mask on top, ready to be used as an etch mask for the underlying chromium layer (see Figure 2B).
3. The resilience of PMMA against plasma etching is quite low; therefore, the resist pattern was transferred into a Cr layer acting as a hard mask for the subsequent structuring of the SiO_2_-layer. Cr was used as mask material because it provides high pattern fidelity and smooth channel sidewalls, which is important for the next SiO_2_ etch step. Etching of the chromium hard mask was performed in a FHR MS-200-2-AE system using a mixture of Cl_2_ (100 sccm) and O_2_ (30 sccm) at a pressure of 32 Pa, a power of 300 W and a chuck temperature of 8 °C for 150 s. The patterning of the SiO_2_ channels was subsequently performed at an ICP Oxford Plasmalab System 100, using a gas flow of 10 sccm CHF_3_ and 18 sccm C_4_F_8_. The etching time was 180 s (see Figure 2C).
4. In the following process step, the remaining resist residues and Cr on top of the SiO_2_ as well as on the channel floors were removed using O_2_ (1850 W) in an R3T STP2020 plasma reactor, and the SiO_2_ surface was passivated using PEG (see Figure 2D).
5. Biofunctionalization of the Au surface with kinesin-1 motor proteins followed just before the experiment (see Figure 2E).

**Figure 3:**
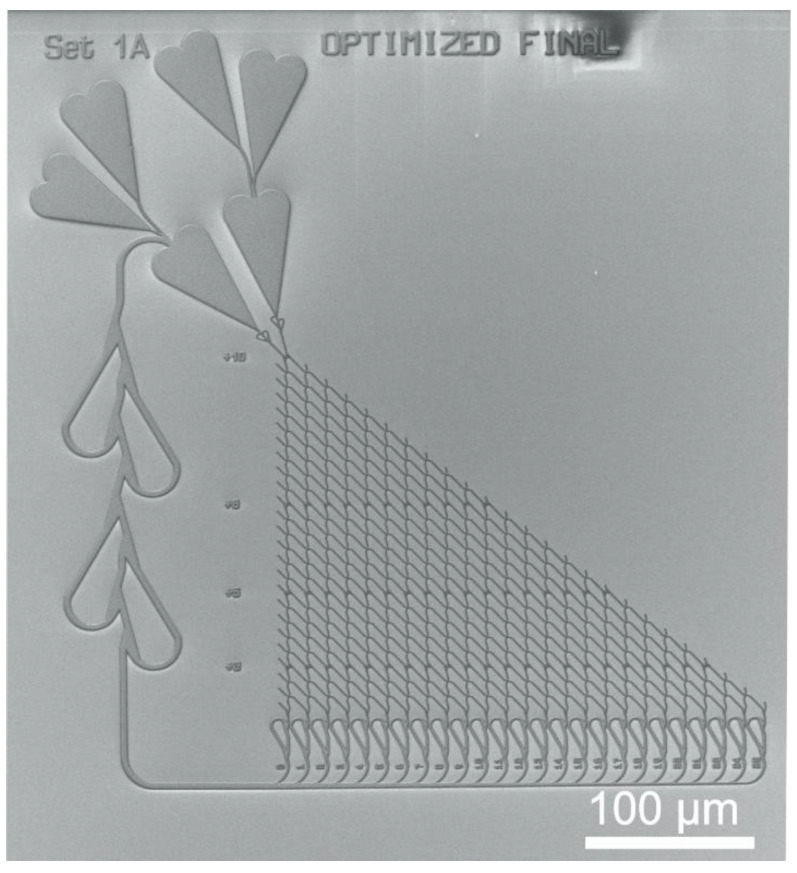
Example of a biocomputational NBC nanochannel network. SEM micrograph of the nanolithographically fabricated NBC network structure designed for the propagation of microtubules driven by kinesin.

Figure 3 shows an SEM micrograph of the nanopatterned channel network with the SSP layout {10, 6, 5, 3}.

The surface passivation, the biological functionalization, and the application of the microtubule gliding motility assays were performed by known procedures [7,25] that were optimized for high motility in the obtained NBC channel system [8].

Briefly, the SiO_2_ surface of the computational chip was passivated with 2-[Methoxy(poly-ethyleneoxy) propyl] tri-methoxysilane] 90% (ABCR, SIM4492.7; 0.23% v/v in toluene·HCl) overnight at room temperature to prevent protein binding anywhere except on the Au floor of the channels. Flow cells were constructed by placing stretched stripes of Parafilm on the chips next to the structures. The channels were closed with a glass coverslip (Menzel, (18 × 18) mm^2^) silanized with PEG as described for the structures above. Flow cells were perfused with casein-containing solution (0.5 mg/mL) in BRB80 solution and left to adsorb for 5 min. Next, 5 μL of kinesin-1 solution (4 nM full-length kinesin-1) was perfused into the flow cells and incubated for another 10 min. Thereafter, a motility solution (1 mM ATP, 20 mM D-glucose, 20 μg/mL glucose oxidase, 10 μg/mL catalase, 10 mM DTT, 10 μM taxol in BRB80) containing rhodamine-labeled, taxol-stabilized microtubules was applied.

Full-length kinesin-1 from Drosophila was expressed in insect cells and purified as described previously [24]. Tubulin was isolated from the porcine brain and subsequently labeled with rhodamine as described previously [26].

The investigation of the motility was performed by recording fluorescence time-lapse movies in an Axiovert 200M inverted optical microscope (Zeiss, Oberkochen, Germany) using a tetra methyl Rhodamine Iso-Thiocyanate filter set (Chroma Technology Corporation. Bellows Falls, VT, USA; Ex 535/50, DM 565, BA 610/75). Time-lapse images were recorded at a rate of 0.5 frames per second with an exposure time of 100 ms using an EMCCD camera (iXon+EMCCD, DU-897E, Oxford Andor, Belfast, UK) in conjunction with Metamorph imaging software (ImageJ-IJ 1.46) (Universal Imaging Corp., Bedford Hills, NY, USA).

### 3.2. Actin-Myosin System

**Figure 4:**
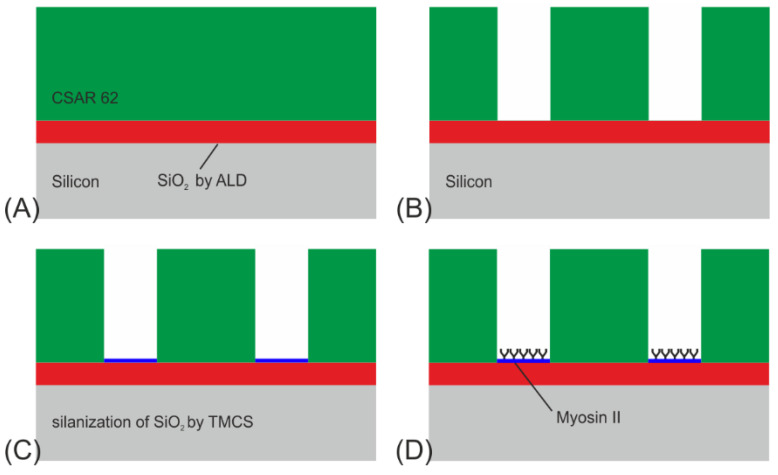
Process flow for NBC nanochannel networks for the actin-myosin system: (**A**) ALD growth of 70 nm SiO_2_, followed by spin coating of 360 nm CSAR 62. (**B**) E-beam lithography (EBL) for patterning the resist. (**C**) Oxygen plasma treatment for 15 s and derivatization of SiO_2_ by CVD of TMCS. (**D**) Functionalization of the SiO_2_ at the channel floors by applying myosin II motors.

The biocomputational network was formed in CSAR 62 resist on a SiO_2_-coated silicon substrate. (Figure 4). For the actin-myosin system, the difference in charge and hydro-phobicity of the channel walls and floors enabled myosin motors to adhere in a motility-promoting manner only to the channel floors [27,28].

The structures were fabricated on a single-side polished 2-inch Si(100) wafer in four steps (see Figure 4A–D):

1. A 70 nm thick SiO_2_ layer was deposited onto the Si-substrate by atomic layer deposition (ALD), using pulses of bisdiethylaminosilane as a precursor in an oxygen plasma. The reason for applying this layer is twofold: to enable surface derivatization by TMCS to modify the surface hydrophobicity, and to enhance the contrast of fluorescently labeled actin filaments. The latter, known as fluorescence interference contrast (FLIC), enables signal enhancement through constructive interference of the emission signals of the fluorophores located on the filaments, excited by either direct light from the light source or light reflected by the Si surface. Subsequently, a layer of CSAR 62 (ALLRESIST AR-P 6200) was spin-coated onto the SiO_2_, to a thickness of around 360 nm and pre-baked at 180 °C for 2 min.
2. The network was patterned by EBL (Raith150) at 50 kV with a dose of 60 µC/cm^2^. The CSAR 62 was then developed for 2 min in O-xylene, rinsed with IPA, and dried with N_2_ gas.
3. To remove any resist residues and to activate the SiO_2_ surface with OH-groups, the devices were ashed in an oxygen plasma (750 W) for 15 s at 5 mbar. Once activated, the surface was derivatized with trimethyl chlorsilane (TMCS) by chemical vapor deposition as described in Ref. [29] at 200 mbar for 64 min at room temperature, providing a water contact angle of app. 75°. The plasma ashing has the additional benefit of making the surrounding resist surfaces and walls negatively charged and hydrophilic, which causes them to adsorb the motors in a non-functional form.
4. Biofunctionalization of the SiO_2_ surface using myosin II motor proteins followed.

Flow cells were built by gluing the device face down to microscope coverslips (No. 0, Menzel-Gläser, Braunschweig, Germany) using two stretches of 60 µm thick double-sided tape. The in vitro motility assays were performed at 22 °C according to a modified protocol previously described [30,31]; 120 µg/mL heavy meromyosin was incubated for 3 min, 1 mg/mL bovine serum albumin (2 min), 50 nM rhodamine-phalloidin-labeled actin filaments (2 min). The ionic strength was 60 mM and the MgATP con-centration was 1 mM.

The motility assays were imaged using a Nikon inverted microscope with a Mercury lamp, an x100 1.4 NA oil immersion objective, and a TRITC filter set (excitation: 532–554 nm, emission: 570–613 nm, dichroic cut-on: 562 nm). The recordings were captured at a rate of 5 frames per second using an EMCCD camera (Hamamatsu C9100) and analyzed with ImageJ [32].

## 4. Results of the EBL Technology Optimizations for NBC and Their Discussion

Biocomputation with biological agents requires several technological preconditions.

- Fabrication of smooth and narrow channels that reliably guide motility and do not allow the filaments to make U-turns [33]
- Fabrication of devices that ensure continuous motility from the source of agents to the pool of possible solutions [34].

### 4.1. Microtubule-Kinesin System 

**Figure 5:**
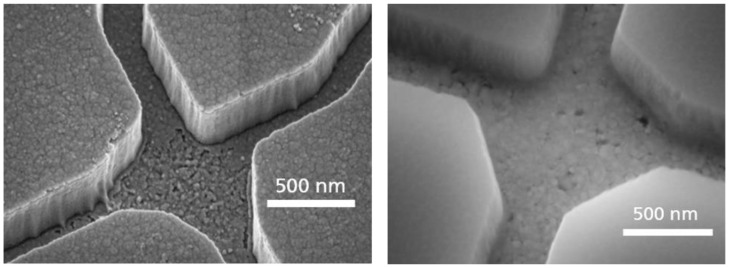
Improvement of the surface roughness of the NBC nanochannel junctions by introducing a chromium layer to protect the gold layer morphology during the reactive ion etch. (**Left**) Rough SiO_2_ sidewalls and Au channel bottom using PMMA for patterning. (**Right**) Smooth channel surface using Cr as a hard mask for SiO_2_ patterning and as an adhesion layer.

In order to achieve high pattern fidelity and smooth channel sidewalls, we evaluated a Cr layer on top of the SiO_2_ layer, acting as a hard mask during the SiO_2_ plasma etch. During this etching step, the Cr layer showed nearly no removal of the flouridic plasma. This resistance also protected the Au layer on the channel floor. The use of Cr hard mask resulted in a high pattern fidelity and smooth channel sidewalls, which is important for the motility of the agents in these channels. The differences in patterning the SiO_2_ by using a standard PMMA EBL resist compared to a Cr hard mask are shown in Figure 5. In the left image of Figure 5, a PMMA mask was used, resulting in almost rough channel sidewalls. By using a Cr hard mask for the SiO_2_ etching process, much smoother sidewalls and the channel bottom could be achieved (see Figure 5 right). These smooth surfaces were necessary to realize the transport of microtubules through the network without their sticking to the sidewalls.

**Figure 6:**
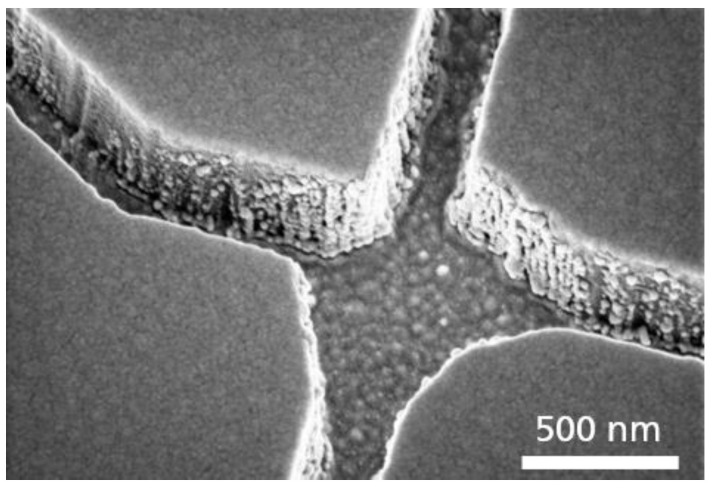
Surface roughness. SEM micrograph of a nanochannel junction with Au redeposited, during the SiO_2_ plasma etching on the SiO_2_ sidewalls when using a titanium instead of a chromium adhesion layer.

Two different adhesion layers (titanium and chromium) between the SiO_2_ and the gold layer were investigated regarding their plasma etch durability during the SiO_2_ patterning. The titanium adhesion layer was used in previous experiments for the fabrication of biocomputational networks [9]. However, during our investigations, the Ti was already attacked and almost removed during the plasma etching of the SiO_2_, so by degrading this Ti layer, the buried Au layer was ablated, resulting in an increase in surface roughness of the channel bottom and of the SiO_2_ sidewalls due to the deposition of the sputtered Au at the SiO_2_ sidewalls (Figure 6).

Concluding from the above, the reliability and the yield of the network structures are superior if Cr is used as an adhesion layer. This protects the Au surface during the SiO_2_ etch and allows to achieve smoother sidewalls. In addition, the chrome can be removed without leaving residues using an O_2_-plasma etch process. 

**Figure 7:**
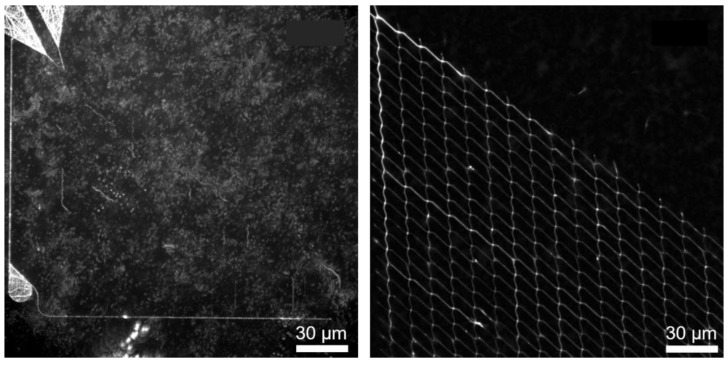
Motility performance of two different prepared NBC networks with the same layout: Ti (**left image**) and Cr (**right image**) were used as adhesion layers between Au and the channel-forming SiO_2_ on top. Both images show the maximum projection of one thousand typical fluorescence micrographs of microtubules moving in the channel network. The white signal indicates areas that were frequently visited by microtubules. In the left image, due to the FLIC effect, only the loading zones (funnels in the left upper corner of the left micrograph) show microtubules close to the Au surface but the nanonetwork is not observable. Only microtubules in the surrounding solution can be observed. In the right micrograph, the nanonetwork is clearly visible, due to the point that the microtubules are also located in the nanochannels near the surface, yielding an increased light intensity (FLIC).

Functionalization tests with the cytoskeletal filaments moving through the network showed that samples with the Ti adhesion layer performed worse in the end. Figure 7 shows the standard deviation projections of timelapse fluorescence micrographs using Fluorescence Interference Contrast Microscopy (FLIC, cf. Ref. [35]). FLIC occurs when fluorescent objects are close to a reflecting surface. The resulting interference between the direct and reflected light leads to an increase in the intensity of the fluorescent object. This means that the filaments close to the surface (due to interaction with the motor proteins appear brighter and more defined [35]. Figure 7 (left) shows the maximum projection of fluorescence micrographs of microtubules, in which no clear path of the microtubules in channels can be distinguished. This indicates that the microtubules are not transported by the motor proteins but are in the solution, i.e., no motility of the microtubules can be observed within the channels for the sample that uses Ti as an adhesion layer. Only motility on the surface within the loading zones could be observed but no moving of microtubules within the channels. Figure 7 (right) shows the maximum projection image of the same network structure that uses Cr as the adhesion layer. There, it can be seen that the microtubules move within the channels through the network. Due to the increase in surface roughness and deposition of the sputtered Au at the SiO_2_ sidewalls, when Ti was used as an adhesion layer, the motor proteins have the possibility to bind not only to the channel bottom but also to the sidewalls, which could lead to the detachment and the unguided motility of microtubules.

Another challenge was the ratio of large structures (loading zones) to small structures (network channels) (see Figure 3) during the dry etch processes. The opening ratio of the areas critically influences the homogeneity and rate during the plasma etching. Due to the good selectivity of the Cr adhesion layer, longer etching times became possible without risking surface degradation of the underlying Au layer.

The flexural rigidity, or persistence length, of the filaments determines their ability to turn around inside the channels and make U-turns [27,36,37]. Microtubules are stiffer than the actin filaments and do not move well through narrow, bent channels. Therefore, it is important to scale the channel sizes to each cytoskeletal system.

**Figure 8:**
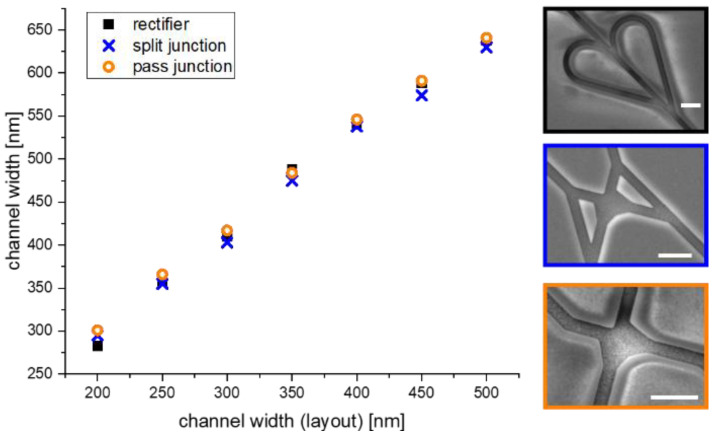
Accuracy of the nanolithographic NBC channel fabrication with respect to channel width. Comparison of the channel broadening of the NBC nanochannels during SiO_2_ plasma etch. The scale bar in each image indicates a length of 1 µm.

The channel width plays an important role in the motility and the behavior of the agents at the junctions; therefore, the process-related broadening of the channel width during SiO_2_ plasma etching was investigated. Seven network structures were fabricated having the same overall layout but with different channel widths ranging from 200 nm up to 500 nm. Subsequently, the channel widths of the different structures (rectifiers, split, and pass junctions) were analyzed using a scanning electron microscope (JSM 7800F) (see Figure 8 right).

The measurements in Figure 8 reveal a linear dependence between the designed width and the resultant structure width at the end of the fabrication process. This finding enabled us to easily scale the designs to the channel sizes necessary to achieve good motility of the cytoskeletal filaments without U-turns. In support of that conclusion, we did not observe a single event where U-turns occur when the approach was tested for the microtubule-kinesin system.

After fabrication, SiO_2_ channel walls for the kinesin-microtubule system were selectively passivated by PEG-silane. This effectively prevents the binding of motor proteins to the channel walls and allows binding to the channel floors, resulting in very good guiding [28,29].

**Figure 9:**
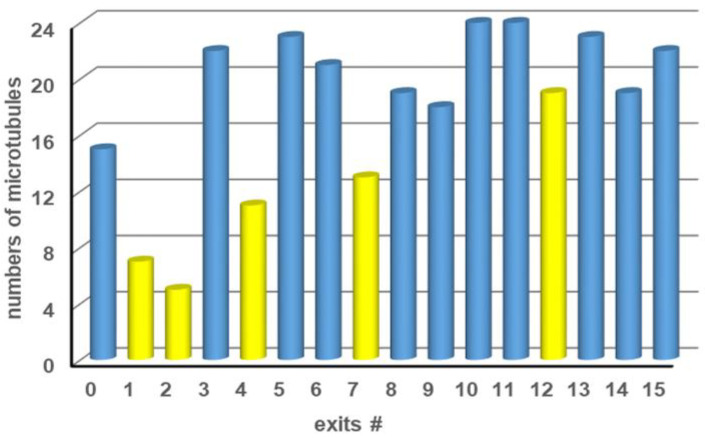
Number of microtubules counted at each exit after exploring a {10, 6, 5, 3} SSP network. Exits where microtubules are expected to arrive are labeled in blue; exits where no microtubules should arrive are labeled in yellow.

The biocomputational network for kinesin-microtubule motility encodes one particular four-variable instance of the SSP {10, 6, 5, 3}. The network was fabricated using the optimized technology and patterning parameters described in the experimental section. Observing microtubules exiting at the first 16 exits of the network (fitting into one field-of-view of our microscope) showed that significantly more microtubules exited at positions corresponding to correct than incorrect solutions (Figure 9). Thus, we were able to solve the specific instance {10, 6, 5, 3} of SSP with twice as many possible solutions compared to [4], using networks fabricated by EBL combined with the biomolecular motor kinesin-1 and their associated cytoskeletal filament microtubules. Please note that almost all errors (filaments exiting at incorrect exits), such as the unanticipated high number of agents in channel 12, result from microtubules that landed in the channels and not from microtubules that made a calculation error during their travel through the network. Microfluidic encapsulation containing hydrodynamic-focusing structures will help to eliminate this source of error and ensure that microtubules can land only in the loading zones of the device and not in the channels of the network. Errors due to wrong turns at pass junctions, which could be relevant in larger networks, could be suppressed by fabricating three-dimensional junctions using two-photon polymerization (TPP) as direct writing technology [38,39], which can—under ideal circumstances—be integrated into the network fabrication using EBL demonstrated here [40].

### 4.2. Actin-Myosin System

Early proof-of-principle networks for the actin-myosin system were relatively inexpensive to fabricate due to their small size [4]. However, the increased dimensions associated with upscaling quickly make fabricating these structures infeasible without process optimization for high throughput. These earlier networks were patterned by electron-beam lithography (EBL) in poly(methyl methacrylate) (PMMA) resist. PMMA requires high beam doses whereas newer resists, such as CSAR 62 (AllResist GmbH, Strausberg, Germany), can provide a comparable resolution at much lower doses, if a suitable developer is selected.

**Figure 10:**
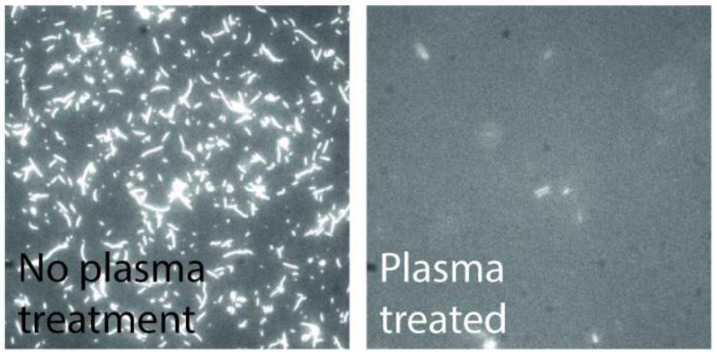
Fluorescence micrographs of actin filaments on CSAR 62. The high motility on CSAR 62 suggests the resist is non-toxic for the actin-myosin system; the motility can be completely suppressed by plasma treatment of the resist.

As the overall device relies on the biological function, it is critical that the polymer used is non-toxic for the particular protein system in use. Furthermore, the polymer also needs to inhibit myosin binding to prevent motility on the walls. We performed in vitro motility assays on CSAR 62 surfaces and observed good motility, comparable to that on TMCS-derivatized SiO_2_ [27]. By treating a CSAR 62 polymer surface with oxygen plasma, we were able to completely suppress the actin filament motility (see Figure 10. These effects in combination make CSAR 62 structures suitable for the actin-myosin system.

**Figure 11:**
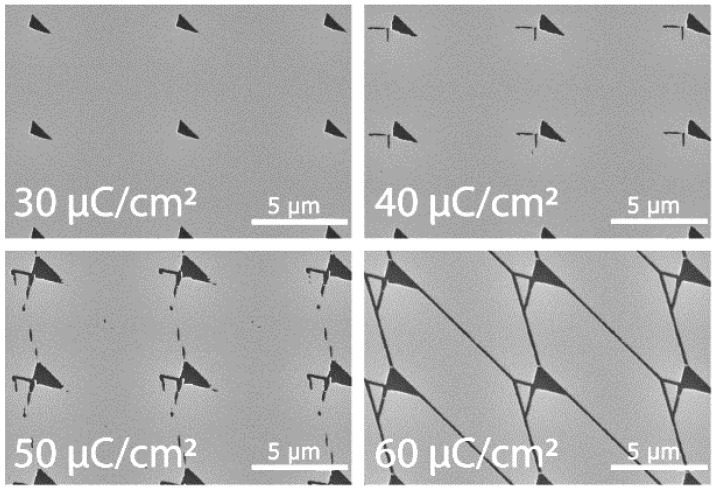
EBL Dose Test. SEM micrograph of EBL dose tests in CSAR 62 resist.

In order to determine what was required for CSAR 62 patterning, four different EBL doses were tested; 30, 40, 50, 60 µC/cm^2^ (with an acceleration voltage 20 kV). All structures were developed in o-Xylene (VWR, Radnor, PA, USA), and we found that it was not possible to achieve complete structures with a dose lower than 60 µC/cm^2^ in our Raith 150 EBL (Raith GmbH, Dortmund, Germany) system (see Figure 11). Structures exposed at 60 µC/cm^2^ instead of 250 µC/cm^2^ required by PMMA can be made roughly four times larger while using the same exposure time.

To enhance the patterning speed, the aperture size of exposure was increased from 10 µm diameter [3] to 20 µm at 20 kV acceleration voltage. This increased the beam current from 0.014 nA to around 0.12 nA, thus, increasing exposure throughput by a factor of 8.5. 

**Figure 12:**
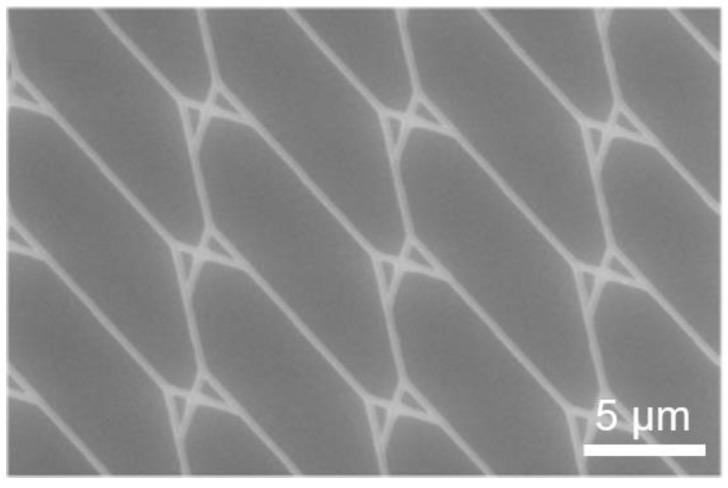
Test pattern to resolve dimension-critical nanostructures. SEM micrographs of a pattern design consisting of split junctions only.

**Figure 13:**
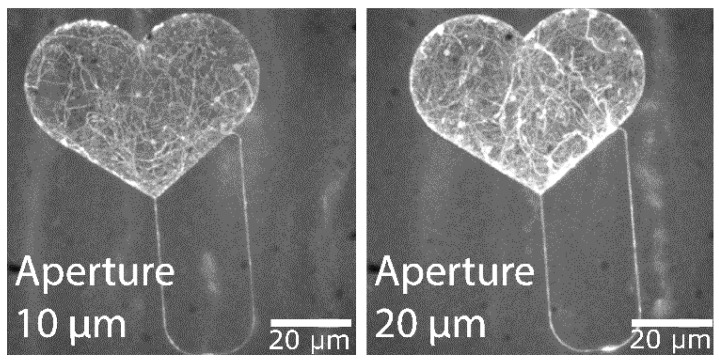
Aperture diameters used for exposure. Fluorescence micrographs showing the maximum intensity projection for 300 frames of actin-myosin motility recording with structures exposed with two different electron beam apertures, 10 and 20 µm.

The structure in Figure 12 was exposed with a 20 µm aperture with no noticeable loss of feature detail compared to the layout. This indicates a stable translation of exposure parameters without an increased effect of electron backscattering, despite the increased aperture size. No U-turns were observed during actin filament motion through nano-channels exposed with this aperture size. A stack of the maximum intensity can be seen in Figure 13, illustrating filament trajectories (bright curved lines) for the structures exposed at 10 µm and 20 µm.

**Figure 14:**
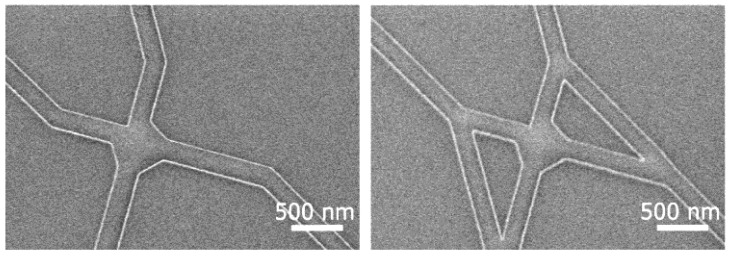
SEM micrographs of the junctions. The images showing well-preserved features even at higher electron-beam-exposure currents, here 0.55 nA, using a 40 µm aperture at 50 kV acceleration voltage.

To further reduce the exposure time, the patterning process was adapted to a Voyager EBL (Raith GmbH, Dortmund, Germany) with a 50 kV acceleration voltage and a 40 µm diameter aperture. This resulted in a beam current of approximately 0.55 nA and an even higher throughput. With this parameter, well-defined features were achieved (see Figure 14). Further optimizations are required to improve the reliability of performance and to ensure that a sufficient number of filaments can reach all exits.

## 5. Summary and Outlook

In summary, we have achieved fast wafer-level nanopatterning with high throughput by using e-beam lithography (EBL) for the scale-up of network-based biocomputation (NBC) applications. For the actin-myosin system, we showed that a four times larger aperture size with simultaneous adjustment of the electron beam current produces no noticeable loss of feature details of the nanochannels and of the performance of the actin motility in these channels. In parallel, the chemically stabilized photo resist CSAR 62 was tested regarding the exposure dose. The experiments reveal a four times less exposure dose than, e.g., PMMA, which results in a four times faster exposure time. In agreement with Ref. [29], we could show that the motility of the CSAR 62 can be suppressed by an oxygen plasma treatment, and it is non-toxic for the motor proteins. This is necessary to achieve mobility on the SiO_2_ surface and not on the resist. These approaches of reducing tremendously the fabrication time and nanodimensional accuracy of patterning enable the fabrication of large (up to 200 mm wafers) networks of nanostructures for the integration of biological molecules. In the case of the microtubule-kinesin system, we improved the smoothness of channel walls and floors to suppress agent sticking to the sidewalls. We also successfully established the processing of biocompatible materials in microfabrication environments at individual batch sizes, allowing individual modifications and iterations of device and wafer layouts.

Perspectively, in conjunction with further improvements in the motor protein expression and the junction layout, the scale-up of biocomputation networks can be extended to larger mathematical problems.

## Data Availability

The data presented in this study are available on request from the authors.
